# Characteristics and Cluster Analysis of 18,030 Sepsis Patients Who Were Admitted to Thailand's Largest National Tertiary Referral Center during 2014–2020 to Identify Distinct Subtypes of Sepsis in Thai Population

**DOI:** 10.1155/2024/6699274

**Published:** 2024-07-30

**Authors:** Phuwanat Sakornsakolpat, Surat Tongyoo, Chairat Permpikul

**Affiliations:** ^1^ Department of Medicine Faculty of Medicine Siriraj Hospital Mahidol University, Bangkok, Thailand; ^2^ Division of Critical Care Department of Medicine Faculty of Medicine Siriraj Hospital Mahidol University, Bangkok, Thailand

## Abstract

**Background:**

This study aimed to investigate the demographic, clinical, and laboratory characteristics of sepsis patients who were admitted to our center during 2014–2020 and to employ cluster analysis, which is a type of machine learning, to identify distinct types of sepsis in Thai population.

**Methods:**

Demographic, clinical, laboratory, medicine, and source of infection data of patients admitted to medical wards of Siriraj Hospital (Bangkok, Thailand) during 2014–2020 were collected. Sepsis was diagnosed according to the Sepsis-3 criteria. Nineteen demographic, clinical, and laboratory variables were analyzed using hierarchical clustering to identify sepsis subtypes.

**Results:**

Of 98,359 admissions, 18,030 (18.3%) had sepsis. Respiratory tract was the most common site of infection. The mean Sequential Organ Failure Assessment (SOFA) score was 4.21 ± 2.24, and the median serum lactate level was 2.7 mmol/L [range: 0.4–27.5]. Twenty percent of admissions required vasopressor. In-hospital mortality was 19.6%. Ten sepsis subtypes were identified using hierarchical clustering. Three clusters (clusters L1–L3) were considered low risk, and seven clusters (clusters H1–H7) were considered high risk for in-hospital mortality. Cluster H1 had prominent hematologic abnormalities. Clusters H3 and H5 had younger ages and significant hepatic dysfunction. Cluster H5 had multiple organ dysfunctions, and a higher proportion of cluster H5 patients required vasopressor, mechanical ventilation, and renal replacement therapy. Cluster H6 had more respiratory tract infection and acute respiratory failure and a lower SpO_2_/FiO_2_ value.

**Conclusions:**

Cluster analysis revealed 10 distinct subtypes of sepsis in Thai population. Furthermore, the study is needed to investigate the value of these sepsis subtypes in clinical practice.

## 1. Introduction

Sepsis is a clinical syndrome that is characterized by a dysregulated immune response to infection, which results in organ dysfunction [1]. Despite continuing advances in critical care medicine, the mortality rate among those who develop sepsis remains high. A worldwide survey of hospital mortality among severe sepsis patients revealed a mortality rate ranging from 17% to 26% [2]. At our center, which is Thailand's largest national tertiary referral center, the mortality rate among severe sepsis and septic shock patients was reported to be 37.9% [3]. Apart from the severity of infection, factors that contribute to higher mortality in sepsis patients include the nature of severe generalized inflammation, persistent immunosuppression, the physical and emotional manifestations associated with postsepsis syndrome [4, 5], and the lack of a specific treatment for sepsis. Trials that aimed to ameliorate altered immune response in sepsis were largely unsuccessful. The overriding reason for this failure is that sepsis is a heterogeneous syndrome that is characterized by a number of clinical and pathophysiological features.

Diagnostic tools for sepsis, such as the Systemic Inflammatory Response Syndrome (SIRS) criteria [6] and the Sepsis-2 and Sepsis-3 diagnostic criteria [1], have been developed and implemented; however, in patients with heterogeneous manifestations, these diagnostic tools do not offer specific clues that suggest or indicate the need for additional specific management. Current research interests and efforts have endeavored to classify sepsis based on demographic, clinical, and laboratory characteristics using machine learning, which is an application of artificial intelligence (AI). Grouping methodologies vary from using empirical clinical characters, such as fever [7] and site of infection [8], to the use of an agnostic/generalized concept [9–13]. Moreover, several trials that are investigating the efficacy of specific treatments for patients with different subtypes of sepsis are currently ongoing.

Most of the previous clustering analyses used data from clinical trials conducted in the United States or from the electronic health records of American patients. In addition to differences in data collection, recording, and availability between medical centers in low-, middle-, and high-income countries, differences also exist relative to the nature of sepsis, such as the source of infection, the causative organisms, and the host setting. These differences between advanced and developing countries suggest that there may also be important differences in sepsis subtypes.

Accordingly, the aim of this study was to investigate the demographic, clinical, and laboratory characteristics of sepsis patients who were admitted to our center during 2014–2020 and to employ cluster analysis of those patient characteristics to identify distinct subtypes of sepsis in Thai population.

## 2. Methods

This retrospective observational study was conducted at the Division of Critical Care of the Department of Medicine, Faculty of Medicine Siriraj Hospital, Mahidol University, Bangkok, Thailand. The protocol for this study was approved by the Siriraj Institutional Review Board (SIRB) on November 4, 2020 (COA no. 850/2563 [IRB4]). Written informed consent to participate was not obtained from included study subjects due to the retrospective, anonymity-preserving nature of this study.

### 2.1. Study Variables

Patient demographic data, sources of infection, clinical characteristics, and laboratory values were collected from our center's electronic medical record system. To label patients with suspected infection, we retrieved drug administration records from our hospital's electronic medical record system by filtering for intravenous antibiotics available at our center. Concerning laboratory parameters, we collected values available within 24 hours of each patient's admission time. If there were multiple measurements during this initial 24-hour interval, the most abnormal value was recorded. The human body system categories and specific laboratory parameters evaluated in this study are listed in Table 1.

### 2.2. Determination of Patients with Sepsis

We used the Sepsis-3 diagnostic criteria to define sepsis in this study. The Sepsis-3 criteria comprise two components, including suspicion of infection and evidence of organ failure. For this study, we defined suspicion of infection as the prescription of intravenous antibiotics at least one dose (as documented in drug dispensing records in any day) during the first 24 hours of admission. For organ failure, we used a Sequential Organ Failure Assessment (SOFA) score [14] of greater than 2 as a cutoff value. A SOFA score was determined for each enrolled patient. The components of the SOFA score and how they are measured are shown in Table 2.

### 2.3. Study Population and Sepsis Patient Selection

To maximize the sensitivity of sepsis diagnosis, we screened all admission data during 2014–2020 from all general medical wards within the Department of Medicine at our center. Admission to a Department of Medicine ward requires a patient age of 16 or older per hospital policy. We then evaluated drug administration records and flagged the cases that received intravenous antibiotics administration for at least one day during hospital admission. This was followed by calculation of the SOFA score using available clinical and laboratory data recorded during the first 24 hours of admission. For missing data, we calculated the SOFA score using only the available variables. No imputation was performed in cases of missing data.

Patients satisfying all of the following criteria were considered to have had sepsis: (1) age 16 years or older; (2) admitted to a general medical ward at Siriraj hospital during 2014–2020; (3) had received at least one dose of intravenous antibiotics; and (4) must have satisfied the Sepsis-3 diagnostic criteria. Patients who were discharged against advice, who left the hospital before being formally discharged from the hospital, or who were referred to other hospitals were excluded. Patients who were missing more than 50% of the data evaluated in this study were also excluded.

### 2.4. Sample Size Calculation

There is currently no standard method for calculating a sample size for a cluster analysis [16], and testing for statistical significance was not performed due to the exploratory nature of this work. We included all patients hospitalized in a general medical ward at our center (sepsis and nonsepsis) during 2014–2020 in the analysis. There were 98,359 admissions during the 7-year study period, and 18,030 (18.3%) of those were determined to have had sepsis.

### 2.5. Statistical Analysis

#### 2.5.1. Baseline Characteristics

Patient demographic, clinical, and laboratory data are given as follows: number and percentage (%) for categorical data; mean plus/minus (±) standard deviation for normally distributed continuous data; and median and [range] for non-normally distributed continuous data. No tests between or among groups or clusters were performed.

#### 2.5.2. Data Preparation for Cluster Analysis

To ensure data quality, we performed quality control for each variable by marking records with invalid data as missing. Missing values for cluster analysis were imputed using the multivariate imputation via chained equations (MICE) algorithm in *R* statistical software version 3.5.0 (the *R* foundation for statistical computing, Vienna, Austria). To facilitate further cluster analysis, we centered and scaled each data variable. This was achieved by subtracting the mean of each column from each row and dividing each data variable by its standard deviation, using the scale function in *R* statistical software.

#### 2.5.3. Cluster Analysis

We performed two types of cluster analysis, including K-means clustering and hierarchical clustering. K-means clustering is a centroid-based clustering method [17]. This method determines the similarity between and among individual participants. Then, the distance between the epicenter of the cluster and each individual member is iteratively calculated to maximize the distance between clusters and to minimize the distance between participants in the cluster. We used the elbow and silhouette methods to determine the optimal number of clusters for K-means clustering. Hierarchical clustering computes the distance between two participants (dissimilarity) and then groups participants who had low distance between the two participants (high similarity). This process is iteratively performed, which results in the development of a cluster of cases with similar predominant characteristics. We used Ward's method to measure cluster distance during the aforementioned process, utilizing the hclust function in *R* statistical software. For hierarchical clustering, we determined the number of clusters by evaluating the cluster dendrogram, where the *y*-axis represents the height. The number of clusters could be obtained by cutting the dendrogram at a specific height. However, there is no consensus method for determining the optimal number of clusters. We determined the number of clusters by inspecting the characteristics of each cluster and considering their clinical implications for further validation.

It should be noted that we first used K-means clustering for our analysis, but the results of that clustering analysis method did not convince us that it could generate convincing and reliable clusters. It has been shown that different clustering analysis methods can generate different results from the same data structure, which suggests that not all clustering methods and data structures are a favorable match. This may have been the reason that we found the K-means method to not be wholly satisfactory. Alternatively, we tried and adopted the hierarchical clustering method for this study given its demonstrated ability to generate reliable and convincing clusters of sepsis subtypes.

#### 2.5.4. Outcome Assessment

To characterize the outcome for each cluster, we established in-hospital mortality as the primary outcome. We obtained the patient's discharge status to determine if the patient was discharged alive or if the patient had expired during hospital admission. Other outcomes included use of mechanical ventilation, use of renal replacement therapy, and use of vasopressors. We determined vasopressor use by obtaining drug administration data during hospital admission, and we filtered our search to flag only norepinephrine, dopamine, and adrenaline. To identify patients who had received mechanical ventilation and/or renal replacement therapy, we used the hospital billing codes in the International Classification of Diseases-Tenth Revision-Clinical Modification (ICD-10-CM) to obtain these data.

## 3. Results

### 3.1. The Characteristics and Prevalence of Sepsis among Hospitalized Patients at Siriraj Hospital

**Figure 1:**
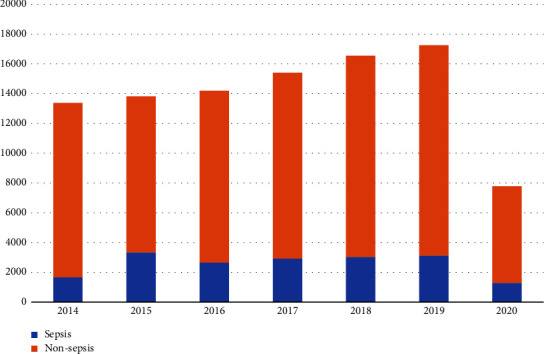
The number of annual hospital admissions compared between nonsepsis patients and sepsis diagnosed according to the sepsis-3 diagnostic criteria during the years 2014 to 2020.

Among the 98,359 admissions to our center during the 7-year study period, 35,996 (36.6%) patients had suspected infection and 18,030 of those satisfied the Sepsis-3 diagnostic criteria. Prevalence of sepsis was 18.3% (18,030/98,359) of all admissions and 50.0% (18,030/35,996) of admissions with suspected infection—all of whom had received intravenous antibiotics for at least one day during their hospital admission. We also investigated the Systemic Inflammatory Response Syndrome (SIRS) criteria as a tool for diagnosing sepsis in this same population. Among 18,030 admissions with sepsis as determined by the Sepsis-3 criteria, only 13,377 admissions had sufficient data to calculate the SIRS score. Of those 13,377 admissions, 6,861 or 51% had an SIRS score of ≥2. After stratification by year of admission, the number of sepsis cases ranged from approximately 2,600 to 3,400 cases per year during 2015–2019, but a lower number of cases was observed in 2014 and 2020 (Figure 1). The drop in both the number of total admissions and the number of sepsis cases during 2020 can most assuredly be attributed to the COVID-19 pandemic. The prevalence of sepsis was steady at 18% during 2016–2019.

### 3.2. Clinical Characteristics and Laboratory Measurements

Among the 18,030 admissions diagnosed with sepsis using the Sepsis-3 diagnostic criteria, the mean age was 66.2 ± 17.4 years and 54.0% were males. The overall mean Elixhauser Comorbidity Index in this study was 13.0 ± 8.21, which corresponds with the 10% rate of hospital mortality reported in a previous study [18]. The overall mean SOFA score was 4.21 ± 2.24, and the overall mean SIRS score was lower than 2 (Table 3). Vital signs included in the calculation of the SIRS score were taken at admission, not at the initial presentation. This factor could have adversely influenced the sensitivity of the SIRS score for detecting sepsis. The overall median serum lactate level was 2.7 mmol/L [range: 0.4–27.5], and 20% of overall admissions required vasopressor. The in-hospital mortality rate was 19.6%.

### 3.3. Site of Infection

Respiratory tract infection was the most frequent diagnosis (5,494 patients, 30.5%). Other sites of infection included genitourinary tract infection (3,203 patients, 17.8%), gastrointestinal infection (3,080 patients, 17.1%), skin and soft tissue infection (860 patients, 4.8%), musculoskeletal infection (335 patients, 1.9%), central nervous system infection (219 patients, 1.2%), eye infection (219 patients, 1.2%), infective endocarditis (102 patients, 0.6%), and other infections (3,811 patients, 21.1%).

### 3.4. Cluster Analysis

**Figure 2:**
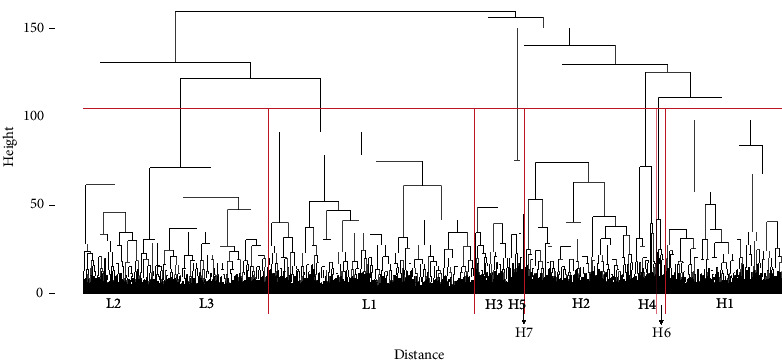
A cluster dendrogram generated using hierarchical clustering that shows 10 clusters (subtypes) of sepsis in red vertical rectangles. The distance between each member of a cluster (*x*-axis) and the height of each cluster (*y*-axis) were used to evaluate the similarity between and among clusters.

By using hierarchical clustering, we created a cluster dendrogram (Figure 2) that shows the distance between individual patients (*x*-axis) and the height (*y*-axis) between clusters. To determine the number of clusters, we cut the dendrogram at different heights, resulting in a varying number of clusters ranging from two to more than twenty-four. The hierarchical nature of this clustering method ensures that increasing the number of clusters does not alter the overall structure; the clusters either aggregate or separate from existing groups, maintaining the same hierarchical characteristics.

We determined the optimal number of clusters by examining the dendrogram and found that having ten clusters provided a balance between understanding the underlying data structure and maintaining low heterogeneity within the clusters. To facilitate the interpretation of the cluster analysis concerning the primary outcome (in-hospital mortality), these ten clusters can be aggregated to form two larger clusters by cutting the dendrogram at its highest separation point.

The two clusters obtained at this level can be categorized based on their in-hospital mortality characteristics. One cluster, termed the low-risk group, comprises clusters with a lower mean in-hospital mortality rate, while the other, termed the high-risk group, consists of clusters with a higher mean in-hospital mortality rate.

Three of ten clusters (clusters L1, L2, and L3) were categorized into the low-risk group, whereas the other seven clusters (clusters H1 to H7) were categorized into the high-risk group. Patients with low risk had fewer comorbidities, a lower serum lactate level, and lower in-hospital mortality, while those in the high-risk group had a lower mean arterial pressure and higher in-hospital mortality (Table 4). Comparing between the clusters in the high-risk and low-risk groups, the high-risk clusters had higher rates of mortality that ranged from 27% to 49%. The high-risk clusters also had a higher mean SIRS score than the clusters in the low-risk group.

Each cluster was found to have its own distinct characteristics. Cluster H1 was the most populated cluster among the high-risk clusters. Patients in this cluster had an average comorbidity score higher than that of all other clusters. They also had higher serum creatinine levels, but the rate of renal replacement therapy was average. Cluster H1 also had higher levels of thrombocytopenia and lower serum albumin levels when compared to the other clusters. Cluster H2 was another highly populated cluster. This group comprised mostly older individuals with average levels of comorbidity. Their outcomes, including mortality, were comparable to those observed in the other clusters in the high-risk group. Cluster H3 included younger individuals with an increased incidence of gastrointestinal infection. Even though this was a younger age cluster, these patients had more comorbid diseases than the patients in other high-risk clusters. They also had higher serum bilirubin and alanine transaminase (ALT). Cluster H4 was found to include older age patients with multiple comorbidities. They had a higher prevalence of respiratory tract infection, and they underwent more renal replacement therapy than the other clusters. Patients in cluster H5 were younger, but harbored multiple comorbidities. They had multiple organ failures, which included respiratory failure requiring mechanical ventilation, the use of renal replacement therapy, and vasopressors, and they had a higher SOFA score, serum lactate level, ALT level, and a prolonged prothrombin time. Cluster H6 was older with some comorbidities. A higher percentage of them had respiratory infections and the lowest SpO_2_/FiO_2_ compared to the other clusters. Similar to the other high-risk clusters, a significant proportion of them needed vasopressors and had high mortality. Cluster H7 was the least populated high-risk cluster in our study (*n*=49), but it had the highest mortality rate. These patients were younger and had less comorbidities compared to the other clusters. The laboratory measurements only showed lower platelet counts and hemoglobin levels. Only small proportion of this cluster used organ support (lower proportion of mechanical ventilation, vasopressors, and renal replacement therapy).

The low-risk clusters were more or less similar with some distinctions. Cluster L1 had male predominance, but Cluster L3 had female predominance. Cluster L2 had higher SOFA scores and serum creatinine levels compared to the other two other low-risk clusters. Their other characteristics were mostly comparable between and among the low-risk clusters.

## 4. Discussion

A retrospective assessment of 98,359 hospital admissions to our center during 2014–2020 revealed 18,030 admissions that fulfilled the Sepsis-3 diagnostic criteria for sepsis. The prevalence of sepsis was 18.3% of patients admitted to general medicine wards. Of those, 20% required vasopressors, and 20% died in the hospital. Ten clusters of sepsis subtypes were identified using demographic, clinical, and laboratory data that was collected during the first 24 hours of admission. Based on cluster characteristics, each cluster could be classified as either high risk or low risk for in-hospital mortality. Three clusters were determined to be low risk, and the other seven were considered high risk for in-hospital mortality. Each of the 10 identified clusters has its own unique set of characteristics and predominant features. This information and further study will improve our ability to anticipate the course of the disease and will help to facilitate treatment strategies that are more specific to each sepsis subtype.

Previously published works of Seymour et al. and Aldewereld et al. [9, 10] examined K-means clustering and hierarchical clustering in sepsis classification. Seymour et al. used a clustering method called K-means clustering to classify patients with sepsis based on demographic, clinical, and laboratory data. This resulted in four clusters of patients that they named alpha, beta, gamma, and delta, and each cluster had unique characteristics. Alpha cluster patients had relatively normal laboratory measurements without organ dysfunction; beta cluster patients were older with multiple comorbidities and poor renal function; gamma cluster patients had higher fever and elevated inflammatory markers; and delta cluster patients had lower mean arterial pressure and significant liver abnormalities. Alternatively, Aldewereld et al. employed hierarchical clustering in patients with early septic shock who were enrolled in the earlier published 2014 Protocolized Care for Early Septic Shock (ProCESS) trial [10]. From the 1,023 patients who were enrolled in the Aldewereld et al. study, the five following sepsis clusters were identified: (1) the low risk for in-hospital mortality cluster 1 (L1 cluster), which was characterized by a predominance of fluid-refractory shock without multiorgan dysfunction; (2) the L2 cluster, which was characterized by a predominance of fluid-responsive shock; (3) the moderate risk for in-hospital mortality cluster (M cluster), which was characterized by a predominance of respiratory failure; (4) the high risk for in-hospital mortality cluster 1 (H1 cluster), which was characterized by a predominance of multiple organ dysfunction; and (5) the H2 cluster, which was characterized by a predominance of hepatobiliary dysfunction and coagulopathy. Other machine learning techniques that have been used to study sepsis include the self-organizing map [11], support vector machine/random forest [12], and latent Dirichlet allocation [13].

To our knowledge, this is the first study in Thailand to perform a cluster analysis of patients with sepsis. To achieve a high sensitivity for detecting sepsis, we collected the electronic medical records of all patients who were admitted to a general medical ward during the study period. We then screened those records for patients who were prescribed intravenous antibiotics for a least one day during their hospital admission period. The SOFA score was then calculated using clinical and laboratory information. We decided on this strategy because it is superior to case detection using a free text search using the term “sepsis” in the electronic medical records or relying on the ICD-10 code for sepsis. Thus, a total number of patients enrolled in our study are much greater than the number of patients enrolled in previous sepsis studies in Thailand [19, 20].

In our study, the severity of sepsis as measured by in-hospital mortality correlated with the cluster hierarchy. As depicted in Figure 2, clusters that are in proximity to other clusters had the comparable in-hospital mortality. The low-risk and high-risk clusters had in-hospital mortality from 9% to 15% and from 27% to 49%, respectively. This implied that the clinical and laboratory features used in clustering indeed capture the underlying drivers of sepsis severity.

The heterogeneity of sepsis is the strongest presumed reason for the failure of therapeutic trials that aimed to mitigate the disease process. Several studies have set forth to subclassify this syndrome to improve understanding, treatment, and outcomes. Studies that, to some degree, dovetail with ours were conducted by Seymour et al. and Aldewereld et al. [9, 10]. Although Seymour and colleagues reported only four subtypes compared to our 10 subtypes, there was some overlap between studies. Our cluster H5 that had the highest SOFA score and marked ALT elevation (mean 2,080 U/L) was similar to their delta cluster [9]. However, any comparison of findings between or among studies relative to cluster characteristics should be performed with caution due to differences in the study population and the data structure, and the fact that some clustering techniques impose arbitrary limits on the number of clusters. Lastly, we decided to use hierarchical clustering instead of K-means clustering, which was the clustering technique used by Seymour et al. because our preliminary analysis using K-means clustering failed to show any distinct structure that we felt could yield reliable clusters. It is known that different methods of cluster analysis rely on different types of data structures, so our observation may simply reflect a suboptimal match between our data structure and the K-means clustering technique. When comparing our work with that of Aldewereld et al. [10], there are some similarities, including their use of hierarchical clustering methodology and their use of subclassification into high- and low-risk groups; however, a number of patients enrolled in their study were much smaller than our study population.

Some sepsis clusters show promise for integration into the clinical development of new therapeutics. Specifically, Cluster H5, characterized by predominant liver dysfunction and coagulopathy, represents a distinct population that may benefit from interleukin-1 receptor antagonists, as suggested by a post hoc analysis of the ProCESS trial [21]. Additionally, Cluster H7, despite its small population size, exhibits the highest in-hospital mortality and features predominant hematologic abnormalities, including thrombocytopenia. Thrombocytopenia in sepsis serves as a marker for poor prognosis, likely resulting from decreased platelet production, consumption, and immune receptor interactions [22]. Patients within this cluster could potentially benefit from a clinical trial evaluating antiplatelet therapy in sepsis [22].

### 4.1. Strengths and Limitations

This study and its findings pave the way for further research to better understand sepsis, and for improved management of sepsis in clinical practice. Moreover, this study identified 10 distinct subtypes of sepsis in Thai population. These findings may lead to the development of a scoring system to clinically classify patients with sepsis. All ten of the sepsis subtypes identified in this study were found to have a distinct clinical course, and this information will help to facilitate improved allocation of healthcare resources to the sepsis subtypes with poorer outcomes. This could lead to a triage system that classifies sepsis patients in clinical practice based on their first 24-hour clinical and laboratory parameters. The construction and validation of this triage system in a prospective trial would be of value in predicting disease severity and may facilitate patient care. The unique cluster characteristics identified in this study can inform clinical trials and prospective sepsis studies. However, identifying patients within these clusters may require the establishment of a dedicated prospective cohort with specific criteria to accurately predict the target population. Additionally, multiple parallel trials with distinct characteristics, conducted as part of an umbrella trial, may be needed to fully explore the implications of these clusters.

Our study also has some mentionable limitations. First, our study's retrospective design rendered it vulnerable to missing or incomplete data, and we did find missing or incomplete data for certain variables in some instances. Although imputation was performed for missing data for the cluster analysis, imputation was not performed for missing data when calculating the SOFA score. Another potential concern is that data specific to vital signs and mental status may reflect measurements taken upon admission, not upon first presentation in the emergency room. This factor may have adversely affected the calculation of the SOFA score since the measurements were taken postresuscitation. Therefore, some sepsis cases may not have been included in the analysis; however, the impact would be small due to the minor contribution of these clinical data in the SOFA score. Second, we did not calculate a sample size for our study because there is not yet any established method for calculating a sample size for a cluster analysis. Third, since the main aim of this study was to investigate whether distinct subtypes of sepsis exist in Thai population or how many distinct subtypes there might be, we did not perform any between or among group or cluster analyses to identify significant differences between groups, nor did we perform any linear regression analyses to identify significantly associated factors. We intend to more deeply evaluate and analyze the data yield from this study in future research projects. Fourth and last, we defined sepsis by both calculating the SOFA score using the data available in the electronic medical record and based on evidence of intravenous antibiotics administration for at least one day during hospital admission. However, some cases of organ failure may have been due to causes other than sepsis. Using the Sepsis-3 diagnostic criteria to define sepsis, so we are reasonably confident that the majority of cases were indeed sepsis. A well-designed prospective study that accounts for the aforementioned limitations is needed to shore up our findings and to further elucidate many of the still unknown characteristics of and mysteries associated with sepsis.

In the era of artificial intelligence, there are some considerations when using or implementing these computational models. By using unsupervised clustering method, the direct interpretability of the model is not easily feasible. The single or simple combination of clinical or laboratory parameters might not explain differences in the clustering result very well. Also, the clinician may find it challenging to understand and doubtful in implementing the model in the clinical practice. Given potential differences in patient characteristics and availability of variables, applying the model into different healthcare systems needs validation. Prospective validation in targeting health system is needed to validate the reliability of the model and to ensure patient safety.

## 5. Conclusion

Cluster analysis revealed 10 distinct subtypes of sepsis in Thai population that were stratified as either low-risk or high-risk for in-hospital mortality. Furthermore, the study is needed to investigate the value and implications of these sepsis subtypes in clinical practice.

## Figures and Tables

**Table 1:** The system categories and specific laboratory parameters evaluated in this study.

System categories	Laboratory parameters
Liver abnormalities	Aspartate transferase
	Alanine transaminase
	Bilirubin

Respiratory abnormalities	Partial pressure of oxygen in arterial blood

Hematologic abnormalities	Hemoglobin
	Platelet count
	International normalized ratio

Renal abnormalities	Sodium
	Chloride
	Blood urea nitrogen
	Creatinine
	Bicarbonate

Inflammation	White blood cell counts
	Erythrocyte sedimentation rate
	C-reactive protein
	Bands

Others	Albumin
	Glucose
	Lactate
	Troponin

**Table 2:** SOFA score components and how each component is measured.

SOFA score components	Description
Respiration (as measured by PaO_2_/FiO_2_)	PaO_2_/FiO_2_ substituted with SpO_2_/FiO_2_ using the following conversions from the prior study [15] (i) PaO_2_/FiO_2_ of 400 corresponds to SpO_2_/FiO_2_ of 400 (ii) PaO_2_/FiO_2_ of 300 corresponds to SpO_2_/FiO_2_ of 315 (iii) PaO_2_/FiO_2_ of 200 corresponds to SpO_2_/FiO_2_ of 235 (iv) PaO_2_/FiO_2_ of 100 corresponds to SpO_2_/FiO_2_ of 148
Coagulation (as measured by platelet count)	
Liver (as measured by total bilirubin)	
Cardiovascular (as measured by mean arterial pressure and use of vasopressors)	
Central nervous system (as measured by Glasgow Coma Scale score)	
Renal (as measured by creatinine level)	
Renal (as measured by urine output)	Omitted due to unavailable data

FiO_2_, fraction of inspired oxygen; PaO_2_, the partial pressure of oxygen in arterial blood; SOFA score, Sequential Organ Failure Assessment score; SpO_2_, arterial oxygen saturation.

**Table 3:** Demographic, clinical, and laboratory characteristics of the overall study population (*N*=18, 030).

Characteristics	Values
Age (years)	66.2 ± 17.4
Male gender	9,740 (54.0%)

*Disease severity scores*	
Elixhauser Comorbidity Index score	13.0 ± 8.21
SIRS criteria score	1.62 ± 1.12
SOFA score	4.21 ± 2.24
Mean arterial pressure	89.0 [38.7–193]
SpO_2_/FiO_2_	462 [126–476]

*Laboratory data*	
Lactate (mmol/L)	2.70 [0.400–27.5]
Creatinine (mg/dL)	1.66 [0.110–39.2]
Bicarbonate (mmol/L)	20.0 [1.00–76.0]
ALT (U/L)	33.0 [1.00–7,790]
Total bilirubin (mg/dL)	1.00 [0.100–58.7]
Prothrombin time (seconds)	15.0 [9.50–246]

*Site/type of infection*	
Respiratory tract infection	5,494 (30.5%)
Genitourinary tract infection	3,203 (17.8%)
Gastrointestinal infection	3,080 (17.1%)
Skin infection	860 (4.8%)
Musculoskeletal infection	335 (1.9%)
CNS infection	219 (1.2%)
Eye infection	219 (1.2%)
Infective endocarditis	102 (0.6%)
Other infections	3,803 (21.1%)

Data presented as mean ± standard deviation, number and percentage (%), or median [range]. ALT, alanine transaminase; CNS, central nervous system; SIRS criteria, Systemic Inflammatory Response Syndrome criteria; SOFA score, Sequential Organ Failure Assessment score; SpO_2_/FiO_2_, arterial oxygen saturation/fraction of inspired oxygen.

**Table 4:** Demographic, clinical, and laboratory characteristics of patient outcomes for the overall study population compared among the 10 sepsis clusters identified in this study.

Characteristics	Overall (*N*=18, 030)	Cluster L1 (*n*=5, 311)	Cluster L2 (*n*=1, 469)	Cluster L3 (*n*=3, 305)	Cluster H1 (*n*=3, 021)	Cluster H2 (*n*=2, 818)	Cluster H3 (*n*=893)	Cluster H4 (*n*=567)	Cluster H5 (*n*=357)	Cluster H6 (*n*=240)	Cluster H7 (*n*=49)
Age (years)	66.2 ± 17.4	64.3 ± 17.5	62.9 ± 15.9	67.6 ± 18.2	63.5 ± 17.5	74.7 ± 14.3	60.2 ± 15.4	70.5 ± 14.8	59.0 ± 18.9	70.8 ± 17.7	54.9 ± 18.0
Male gender	9,740 (54.0%)	4,850 (91.3%)	848 (57.7%)	24 (0.7%)	1,422 (47.1%)	1,450 (51.5%)	488 (54.6%)	278 (49.0%)	224 (62.7%)	125 (52.1%)	31 (63.3%)
ECI score	13.0 ± 8.21	12.2 ± 7.96	10.1 ± 5.7	10.8 ± 6.98	17.1 ± 9.02	12.3 ± 7.77	16.0 ± 8.29	16.8 ± 8.13	16.7 ± 9.47	13.3 ± 7.94	5.00 ± 5.18
RT infection	5,494 (30.5%)	1,470 (27.7%)	339 (23.1%)	832 (25.2%)	1,043 (34.5%)	1,169 (41.5%)	170 (19.0%)	226 (39.9%)	112 (31.4%)	124 (51.7%)	9 (18.4%)
GU infection	3,203 (17.8%)	685 (12.9%)	178 (12.1%)	786 (23.8%)	546 (18.1%)	683 (24.2%)	105 (11.8%)	120 (21.2%)	48 (13.4%)	50 (20.8%)	2 (4.1%)
GI infection	3,080 (17.1%)	813 (15.3%)	163 (11.1%)	529 (16.0%)	717 (23.7%)	392 (13.9%)	284 (31.8%)	99 (17.5%)	37 (10.4%)	37 (15.4%)	9 (18.4%)
SIRS criteria	1.62 ± 1.12	1.49 ± 1.10	1.22 ± 1.13	1.31 ± 0.98	2.13 ± 1.10	1.84 ± 1.08	1.36 ± 1.06	1.70 ± 1.06	1.97 ± 1.15	2.67 ± 0.96	2.48 ± 0.87
SOFA score	4.21 ± 2.24	3.60 ± 1.72	4.63 ± 1.60	3.62 ± 1.71	4.75 ± 2.49	4.55 ± 2.56	5.28 ± 2.70	4.59 ± 2.62	6.11 ± 3.31	5.04 ± 2.89	4.41 ± 2.13
MAP (mmHg)	89.0 [38.7–193]	90.0 [42.3–190]	103 [48.7–182]	90.0 [42.7–160]	83.0 [39.3–148]	87.7 [38.7–171]	87.0 [48.7–193]	86.7 [40.7–142]	86.7 [42.3–163]	84.3 [43.0–135]	85.5 [67.7–114]
SpO_2_/FiO_2_	462 [126–476]	467 [129–476]	467 [141–476]	467 [136–476]	457 [130–476]	457 [126–476]	467 [137–476]	460 [220–476]	467 [137–476]	379 [190–443]	452 [237–476]
Lactate (mmol/L)	2.70 [0.40–27.5]	2.30 [0.50–27.6]	2.00 [0.40–27.3]	2.00 [0.50–31.7]	3.10 [0.50–27.5]	3.05 [0.50–46.6]	2.40 [0.50–31.5]	3.50 [0.50–45.9]	7.95 [0.90–42.5]	4.30 [0.70–23.7]	3.60 [0.90–28.2]
Creatinine (mg/dL)	1.66 [0.11–39.2]	1.42 [0.11-11.2]	8.66 [0.39-39.2]	1.56 [0.13-13.9]	1.53 [0.12-15.7]	1.47 [0.12-12.9]	1.02 [0.20–22.4]	2.21 [0.24-13.7]	2.52 [0.24-16.5]	1.74 [0.13-10.5]	1.23 [0.42-3.56]
Bicarbonate (mmol/L)	20.0 [1.00–76.0]	21.0 [1.00–49.0]	19.0 [1.00–37.0]	20.0 [2.00–41.0]	17.0 [2.00–39.0]	20.0 [1.00–76.0]	20.0 [4.00–39.0]	18.0 [4.00–41.0]	14.0 [2.00–35.0]	18.0 [4.00–38.0]	19.0 [4.00–31.0]
ALT (U/L)	33.0 [1.00–7790]	33.0 [1.00–1280]	18.0 [1.00–877]	27.0 [2.00–1390]	33.0 [1.00–1860]	30.0 [3.00–1430]	72.0 [6.00–1570]	27.0 [5.00–3850]	2080 [123–7790]	26.0 [3.00–644]	36.0 [7.00–2920]
Total bilirubin (mg/dL)	1.00 [0.10–58.7]	1.03 [0.10–22.3]	0.47 [0.10–8.43]	0.74 [0.10–17.2]	1.28 [0.10–29.3]	0.70 [0.10–18.3]	19.90 [2.70–58.7]	1.20 [0.10–57.9]	2.30 [0.20–25.3]	0.88 [0.15–19.3]	0.81 [0.27–6.05]
Prothrombin time (sec)	15.0 [9.50–246]	14.4 [9.50–63.1]	13.1 [9.50–49.7]	13.9 [9.50–46.8]	16.4 [9.60–68.8]	15.1 [10.1–80.0]	17.8 [9.90–113]	63.8 [19.7–246]	27.0 [10.6–108]	17.9 [10.5–77.4]	17.2 [12.3–158]
Albumin (g/dL)	2.90 [0.60–6.00]	3.10 [0.70–5.40]	3.30 [0.80–6.00]	3.00 [1.00–5.00]	2.50 [0.60–4.90]	2.80 [0.90–4.80]	2.60 [0.70–4.70]	2.80 [1.00–5.00]	2.90 [0.60–4.90]	2.60 [0.90–4.40]	2.80 [1.70–3.80]
Hemoglobin (g/dL)	9.20 [1.00–24.3]	9.60 [2.80–24.3]	9.00 [3.50–16.4]	9.10 [2.40–17.4]	8.30 [1.00–16.5]	9.60 [2.00–23.6]	9.20 [2.50–17.5]	9.40 [2.90–17.4]	10.0 [1.80–20.1]	9.20 [2.70–18.9]	6.40 [2.60–10.5]
Platelet count (/*μ*L)	136 [1.00–2.15]	135 [1.00–2150]	180 [2.00–582]	129 [1.00–650]	91.0 [1.00–987]	157 [1.00–835]	206 [1.00–864]	153 [1.00–593]	107 [3.00–531]	141 [2.00–994]	31.0 [7.00–735]

*Outcomes*											
Vasopressor use	3,614 (20.0%)	898 (16.9%)	113 (7.7%)	550 (16.6%)	807 (26.7%)	713 (25.3%)	139 (15.6%)	154 (27.2%)	148 (41.5%)	86 (35.8%)	6 (12.2%)
Mechanical ventilation	1,849 (10.3%)	430 (8.1%)	127 (8.6%)	222 (6.7%)	332 (11.0%)	497 (17.6%)	42 (4.7%)	71 (12.5%)	80 (22.4%)	44 (18.3%)	4 (8.2%)
RRT	989 (5.5%)	189 (3.6%)	76 (5.2%)	131 (4.0%)	219 (7.2%)	157 (5.6%)	53 (5.9%)	61 (10.8%)	74 (20.7%)	26 (10.8%)	3 (6.1%)
In-hospital mortality	4,010 (22.2%)	802 (15.1%)	133 (9.1%)	481 (14.6%)	995 (32.9%)	877 (31.1%)	244 (27.3%)	195 (34.4%)	151 (42.3%)	108 (45.0%)	24 (49.0%)

Data presented as mean ± standard deviation, number and percentage (%), or median [range]. ALT, alanine transaminase; ECI score, Elixhauser Comorbidity Index score; GI, gastrointestinal; GU, genitourinary; H, high-risk group; *L*, low-risk group; MAP, mean arterial pressure; RRT, renal replacement therapy; RT, respiratory tract; SIRS criteria, Systemic Inflammatory Response Syndrome criteria; SOFA score, Sequential Organ Failure Assessment score; SpO_2_/FiO_2_, arterial oxygen saturation/fraction of inspired oxygen.

## Data Availability

Researchers may contact the corresponding author for data sharing requests, after approval of a planned analysis protocol. The anonymized participant data will be made available within three months after the publication of the article.

## Supplementary Materials

Supplementary Materials STROBE Statement—checklist of items that should be included in reports of observational studies.
